# When Stress Gets on Your Nerves: Associations Among Stress Severity, Sleep, and Headache in Daily Life

**DOI:** 10.1093/geroni/igaa057.2175

**Published:** 2020-12-16

**Authors:** Susanna Joo, Sun Ah Lee, Hye Won Chai, Hey Jung Jun, David Almeida

**Affiliations:** 1 Yonsei University, Seoul, Republic of Korea; 2 The Pennsylvania State University, University Park, Pennsylvania, United States

## Abstract

The present study aims to examine whether duration of sleep moderates the relationship between daily stressor severity and headache severity and test age differences in these associations. We used the second wave of the Midlife in the United States (MIDUS) study and its subproject, the National Study of Daily Experiences (N=1,590). Stress severity was measured based on the respondents’ perception of reported stressors, and headache severity was indicative of subjective rating of the symptom. Multilevel analysis results showed significant interaction effects between concurrent-day stressor severity and duration of sleep. Specifically, stressor severity was associated with more severe headache when people slept for shorter amount of time the same day. There were no differences by age in these associations. These results suggest that shorter-than-usual sleep may aggravate the intensity of headache on stressful days in daily life.

